# Exercise Induced-Cytokines Response in Marathon Runners: Role of ACE I/D and BDKRB2 +9/-9 Polymorphisms

**DOI:** 10.3389/fphys.2022.919544

**Published:** 2022-09-02

**Authors:** Ana Paula Renno Sierra, Bryan Steve Martínez Galán, Cesar Augustus Zocoler de Sousa, Duane Cardoso de Menezes, Jéssica Laís de Oliveira Branquinho, Raquel Leão Neves, Júlia Galanakis Arata, Clarissa Azevedo Bittencourt, Hermes Vieira Barbeiro, Heraldo Possolo de Souza, João Bosco Pesquero, Maria Fernanda Cury-Boaventura

**Affiliations:** ^1^ School of Physical Education and Sport, University of São Paulo, São Paulo, Brazil; ^2^ Interdisciplinary Post-Graduate Program in Health Sciences, Institute of Physical Activity and Sports Sciences, Cruzeiro do Sul University, São Paulo, Brazil; ^3^ Department of Biophysics, Federal University of Sao Paulo, São Paulo, Brazil; ^4^ Emergency Medicine Department, LIM-51, University of São Paulo, São Paulo, Brazil

**Keywords:** angiotensin-converting enzyme, endurance exercise, myokines, bradykinin B2 receptor, polymorphism

## Abstract

Renin-angiotensin system (RAS) and kallikrein-kinin system (KKS) have a different site of interaction and modulate vascular tone and inflammatory response as well on exercise adaptation, which is modulated by exercise-induced cytokines. The aim of the study was to evaluate the role of ACE I/D and BDKRB2 +9/−9 polymorphism on exercise-induced cytokine response. Seventy-four male marathon finishers, aged 30 to 55 years, participated in this study. Plasma levels of exercise-induced cytokines were determined 24 h before, immediately after, and 24 h and 72 h after the São Paulo International Marathon. Plasma concentrations of MCP-1, IL-6 and FGF-21 increased after marathon in all genotypes of BDKRB2. IL-10, FSTL and BDNF increased significantly after marathon in the genotypes with the presence of the −9 allele. FSTL and BDNF concentrations were higher in the −9/−9 genotype compared to the +9/+9 genotype before (*p* = 0.006) and after the race (*p* = 0.023), respectively. Apelin, IL-15, musclin and myostatin concentrations were significantly reduced after the race only in the presence of −9 allele. Marathon increased plasma concentrations of MCP1, IL-6, BDNF and FGF-21 in all genotypes of ACE I/D polymorphism. Plasma concentrations of IL-8 and MIP-1alpha before the race (*p* = 0.015 and *p* = 0.031, respectively), of MIP-1alpha and IL-10 after the race (*p* = 0.033 and *p* = 0.047, respectively) and VEGF 72 h after the race (*p* = 0.018) were lower in II homozygotes compared to runners with the presence of D allele. One day after the race we also observed lower levels of MIP-1alpha in runners with II homozygotes compared to DD homozygotes (*p* = 0.026). Before the marathon race myostatin concentrations were higher in DD compared to II genotypes (*p* = 0.009). Myostatin, musclin, IL-15, IL-6 and apelin levels decreased after race in genotypes with the presence of D allele. After the race ACE activity was negatively correlated with MCP1 (r = −56, *p* < 0.016) and positively correlated with IL-8, IL-10 and MIP1-alpha (r = 0.72, *p* < 0.0007, r = 0.72, *p* < 0.0007, r = 0.47, *p* < 0.048, respectively). The runners with the −9/−9 genotype have greater response in exercise-induced cytokines related to muscle repair and cardioprotection indicating that BDKRB2 participate on exercise adaptations and runners with DD genotype have greater inflammatory response as well as ACE activity was positively correlated with inflammatory mediators. DD homozygotes also had higher myostatin levels which modulates protein homeostasis.

## Introduction

Renin-angiotensin system (RAS) and kallikrein-kinin system (KKS) have a different site of interaction and modulate vascular tone and inflammatory response as well on exercise adaptation (Su, 2014; Cabello-Verrugio et al., 2015; Frantz et al., 2018; Evangelista, 2020). The activation of classical RAS pathway promote increase on angiotensin-converting enzyme (ACE) activity, which catalyzes the conversion of angiotensin I (Ang-I) into angiotensin II (Ang-II) and bind to AT1 and AT2 receptors (AT1R and AT2R) resulting in vasoconstriction, inflammation, sympathetic activation, cell proliferation and angiogenesis (AT1R) or vasodilation, anti-inflammatory, antifibrotic and antiproliferative and antiangiogenic response (AT2R) (Arazi et al., 2021; Bekassy et al., 2021). ACE also degrades bradykinin (BK) released by KKS, which binds to B2 receptor (B2R) and induces inflammation, vasodilation and mitochondrial respiration. Moreover, AT1R and AT2R may form heterodimers with B2R, increasing nitric oxide (NO) production. Controversially, BK signaling via B2R induces renin expression (Lau et al., 2020; Bekassy et al., 2021). The vascular and inflammatory response influences ischemia and reperfusion induced by exercise in different tissues (Rabinovich-Nikitin and Kirshenbaum, 2018; Szabó et al., 2020).

The role of *ACE* I/D and *BDKRB2* +9/-9 polymorphisms have been investigated on performance, muscle adaptations and injury (Alvarez et al., 2000; Tsianos et al., 2010; Baumert et al., 2016; Papadimitriou et al., 2018; Sierra et al., 2019; John et al., 2020; Massidda et al., 2020; Varillas-Delgado et al., 2021). *ACE* gene polymorphism (rs1799752) is a well-known genetic variation characterized by the deletion (D) or insertion (I) of 287 base pairs (bp) fragment in 17q23 chromosome (Rigat et al., 1990). II genotype has been associated to an improvement in oxidative metabolism and capillary perfusion (Dietze and Henriksen, 2008; Vaughan et al., 2013; Vaughan et al., 2016), while DD genotype with greater muscle power phenotypes after resistance exercise (Erskine et al., 2014; Freire et al., 2015) and muscle damage protection (Amir et al., 2007; Sierra et al., 2019; Massidda et al., 2020). *BDKRB2* +9/-9 polymorphisms (rs5810761) are characterized by the presence of a nine-base pairs (bp) repeat (+9) or the absence of 9 bp (-9) repeat in exon one of BDKRB2 gene resulting in higher expression of BDKRB2 in -nine to nine homozygotes. Blood flow and vascular conductance promoted by exercise training appear to be greater in *BDKRB2* -9/-9 homozygotes (Alves et al., 2013).

Many of exercise-induced cytokines are modulated by shear stress, metabolic, oxidative and reticulum endoplasmic stress and mitochondrial dysfunction as the result of inflammation and ischemia/reperfusion (Pritchett et al., 2009; Whitham and Febbraio, 2016; Safdar and Tarnopolsky, 2018; Piccirillo, 2019; Bay and Pedersen, 2020; Laurens et al., 2020). More than 650 exercise-induced cytokines have been described to modulate properties essential to tissue regeneration such as protein synthesis or degradation, proliferation and differentiation of myoblasts, activation of satellite cells, organization and remodeling of the extracellular matrix, angiogenesis, autophagy, mitophagy and mitochondrial biogenesis (Whitham and Febbraio, 2016; Safdar and Tarnopolsky, 2018; Piccirillo, 2019; Bay and Pedersen, 2020; Laurens et al., 2020). Exercise-induced cytokines may be released by myocytes, satellite cells, endothelial cells, hepatocytes, residential macrophages, and fibroblasts, affecting skeletal and cardiac muscle as well as blood vessels, adipose tissue and brain (Hoffmann and Weigert, 2017; Lee and Jun, 2019; Laurens et al., 2020). Recently, our group demonstrated the increase of brain-derived neurotrophic factor (BDNF), fibroblast growth factor 21 (FGF-21), follistatin (FSTL) and decorin, as well as a decrease of myostatin, musclin and apelin after the race (de Sousa et al., 2021).

Therefore, the aim of present study was to evaluate the role of ACE I/D and BDKRB2 +9/-9 polymorphisms on exercise-induced cytokines after a marathon race. The identification of exercise-induced peptides in different genotypes may contribute to elucidating the importance of RAS and KKS on exercise adaptations.

## Materials and Methods

### Subjects

The study included seventy-four amateur Brazilian male marathon finishers (aged 30 to 55 years). The volunteer’s recruitment was performed in collaboration with the São Paulo International Marathon Organization (2017 and 2018, Yescom, BRA), which sent an e-mail for all marathon runners registered in the São Paulo International Marathon (2017 and 2018), inviting volunteers to apply for research participation. Then, researchers randomly contacted volunteers to check and confirm interest and availability to participate in the research. They engaged in training sessions completing at least 30 km per week and have participated in one or more marathon or half marathon. Individuals with cardiovascular, pulmonary or kidney injury, as well as including systemic arterial hypertension, kidney, liver, inflammatory or neoplastic diseases or use of alcohol or drugs were excluded from the study.

The study was approved by the research Ethics Committee of Cruzeiro do Sul University, Brazil (Permit Number: 3.895.058) in accordance with the declaration of Helsinki. All volunteers gave written informed consent before participating of the study.

### Anthropometric Data

Measurements of body weight (kg) and height (cm) were performed using an electronic digital scale platform (marte^∗^, Sao Paulo, SP, Brazil). Body mass index (BMI) was calculated according to the formula weight (kg)/height (m^2^), and the International Society for Advancement of Kinanthropometry (ISAK) was considered to these measurements. The body composition was evaluated in fasting state by bioimpedance analysis (Biodynamics Corporation, United States, 310e) 1 day before the marathon race.

### Cardiopulmonary Exercise Test

The cardiopulmonary exercise test (CPET) was performed between three to twenty-one days before the marathon race by progressive treadmill test (TEB Apex 200, TEB, São Paulo, Brazil, speed 0–24 km/h, grade 0%–35%), and a medical history. During the running test, the volunteers were monitored with a standard 12-lead computerized electrocardiogram (TEB^∗^, ECG São Paulo, Brazil) to check for possible cardiac variations during exertion. The participants attended the laboratory, and a progressive treadmill test was performed with a constant incline of 1% and an initial speed of 8 km h^−1^, increasing 1 km h^−1^ every 1 min until voluntary exhaustion. The respiratory gas exchange was monitored through an open circuit and automatic, indirect calorimetry (Quark CPET, COSMED^∗^, Rome, Italy). The VO_2_ max of the subjects was classified according to the American College of Sports Medicine (Thompson et al., 2013). All runners recruited completed the International Marathon of São Paulo 2017 (40 runners) and 2018 (34 runners).

### Blood Sampling

Blood collection (20 mL) from fasting runners with at least 12 h without physical activity was performed 24 h before, 24 h and 72 h after the marathon from the antecubital vein at the Institute of Physical Activity and Sports (Cruzeiro do Sul University). Blood samples were immediately centrifuged at 4°C, 400 g, for 10 min to obtain plasma samples (10 mL, vacuum tubes containing ethylenediaminetetraacetic acid, EDTA, 1 mg/mL) and after 30 min at room temperature to obtain serum samples (5 mL, vacuum dry siliconized tube). The samples were stored at −80°C for later analysis of cytokines-induced by exercise at University of São Paulo. The others blood samples (5 mL, vacuum tubes containing ethylenediaminetetraacetic acid, EDTA, 1 mg/mL) were kept on ice by approximately 2 h and then sent to Federal University of São Paulo for genetic analyses. Immediately after the race, blood samples (20 mL) from fed runners were maintained on ice by approximately 2 h at the International Marathon of São Paulo (competition venue, close to finish line) and then sent to Cruzeiro do Sul University (15 mL) for serum and plasma separation and stored and Federal University of São Paulo (5 mL) for genetic analyses.

### Marathon Race

The São Paulo International Marathon (2017, 2018) started at 07:30 a.m. on April 9 and April 8, respectively. Fluid ingestion was allowed *ad libitum* during the race. Water was available every 2–3 km on the running course; sports drinks were available at 12 km, 21.7 km, 33 km, and 42 km; and a carbohydrate source was available at 28.8 km. The weather parameters between 07:00 a.m. and 02:00 p.m. were as follows: average temperature, 19.8°C (2017) and 19.9°C (2018); average relative humidity, 72.8% (2017) and 87.7% (2018) (National Institute of Meteorology, Ministry of Agriculture, Livestock, and Supply).

### Determination of Cytokines Induced by Exercise

The levels of some interleukins (IL-1ra, IL-4, IL-10), tumor necrosis factor alpha (TNF-α), vascular endothelial growth factor (VEGF), FGF-2, interferon-gamma (IFN-gamma), macrophage inflammatory protein-1 (MIP-1) and monocyte chemoattractant protein-1 (MCP-1) markers were determined using the MILLIPLEX^∗^ Human Chemokine/Cytokine Magnetic Bead Panel technique (HCYTOMAG -60K, EMD Millipore Corporation). Plasma levels of apelin, irisin, BDNF, myostatin, musclin, FSTL, IL-6, IL-15 and FGF-21 were also determined using the MILLIPLEX^∗^ Human Myokine magnetic bead panel techinque (HCYTOMAG-56K, EMD Millipore Corporation, MA, United States).

MILLIPLEX® Human Myokine/Chemokine/Cytokine Magnetic Bead Panel technique is an immunoassay on the surface of fluorescent-coded magnetic beads (MagPlex®-C microspheres) each of which is coated with a specific capture antibody. Luminex® xMAP® technology uses proprietary techniques to internally color-code microspheres with two fluorescent dyes. After an analyte from a sample is captured by the bead, a biotinylated detection antibody is incorporated on assay follow reaction with Streptavidin-PE conjugate. The Luminex®analyzer (MAGPIX®) integrates lasers, optics, advanced fluidics and high-speed digital signal processors of capture and detection components with the speed and efficiency of magnetic beads.

The intra-assay precision (mean coefficient variation percentage) was performed as described by the manufacturer’s protocol, which was <3% for IL-1ra, IL-6, IL-10, IL-15, FGF-2, IFN-gamma, MIP-1 and <10% for TNF-α, VEGF, MCP-1, apelin, irisin, BDNF, myostatin, musclin, FSTL, FGF-21.

### ACE Enzymatic Activity Assay

ACE activity was determined in serum samples on buffer 100 mM Tris-HCl, 50 mM NaCl and 10 µM ZnCl2, pH 7.0, using 10 µM of fluorogenic substrate Abz-FRK (Dnp)P-OH. To confirm the specificity of the assay, samples were incubated with 10 µM of lisinopril inhibitor. The emitted fluorescence (*λ*_ex_ = 320 nm and *λ*_em_ = 420 nm) was monitored every 1 min at 37°C for 30 min in a microplate reader (Synergy H1, BioTek).

### Genetic Analysis of ACE and BDKRB2 Polymorphisms

To determine ACE (I/D alleles) and BDKRB2 (+9/−9) polymorphisms, genomic DNA (gDNA) was extracted from blood samples collected in tubes containing EDTA (ethylenediamine tetraacetic acid) using Chelex®100 resin (Sigma Aldrich) and Proteinase K, according to manufacturer’s instructions. Microtubes containing blood samples were diluted in ultrapure water (1:20) and homogenized in room temperature for 6 min alternately every 2 min. The samples were centrifuged at 10,000 x g for 2 min, the supernatant was discarded and the washing process was repeated twice until the pellet contained fewer red blood cells. Subsequently, Chelex 20% and Proteinase K (10 mg/mL) were added into the microtubes containing the samples and incubated at 56°C for 30 min with constantly shaking, followed by another incubation at 95°C for 8 min. Finally, the samples were centrifuged at 14,000 x g for 10 min and the supernatant containing the gDNA was transferred to a sterile microtube. The quantification of gDNA was determined using NanoDrop One (Thermo Fisher Scientific) on absorbance wavelength at 260 nm.

*ACE* insertion (I) or deletion (D) variants were screened by a polymerase chain reaction (PCR) using an antisense primer (5′-CTG GAG ACC ACT CCC ATC CTT TCT-3′) and sense primer (5′-GAT GTG GCC ATC ACA TTC GTC AGA T-3′). The PCR product resulted in a 490 bp (I) and 190 bp (D) fragment analyzed on a 2% agarose gel stained with SYBR^∗^ Safe DNA gel stain (Invitrogen, Massachusetts, United States).

The presence or absence of repeated sequence of nine nucleotides of the *BDKRB2* polymorphisms were screened by a polymerase chain reaction (PCR) using a sense primer (5′- TCT GGC TTC TGG GGC TCC GAG -3′) and an antisense primer (5′- AGC GGC ATG GCA CTT CAG T -3′). The PCR product resulted in 89 bp (+9) and 80 bp (-9) fragment analyzed on a 4% agarose gel stained with SYBR^∗^ Safe DNA gel stain (Invitrogen, Massachusetts, United States).

### Statistical Analyses

Data are reported as mean ± SEM. Statistical analyzes were performed using GraphPad Prism (GraphPad Prism version 9). Sample size was estimated based on the average of amateur athletes between 30 and 55 years old registered in the São Paulo International Marathon (around 4000 marathon runners), considering confidence level of 95% and confidence interval of 10%, resulting in 75 samples. General characteristics and myokines analyses were performed with 74 samples. Chemokine/cytokines analyses were performed with 41–42 samples, increasing the confidence of interval from 10 to 15%. After the presupposition of normality by the Shapiro-Wilk test, the comparison of the general and training characteristics between the genotypes (II vs. ID vs. DD and -9/-9 vs. + 9/-9 vs. +9/+9) was performed using ordinary two-way ANOVA. Statistical analyzes were evaluated using repeated measures bidirectional ANOVA (time) with Geisser-Greenhouse correction for sphericity and Sídák post-test for multiple comparisons between before and immediately after, 24 and 72 h after the marathon race was applied, as well multiple comparison between genotypes (II vs. ID vs. DD and -9/-9 vs. +9/-9 vs. +9/+9). Correlations between ACE activity and cytokines-induced by exercise were performed by Spearman. General characteristics were evaluated by ANOVA one way test and Tukey test for multiple comparison. Statistical significance was accepted at e level of *p* < 0.05 in all analyses.

## Results

### General Characteristics

In this study, the genotypic frequencies for the BDKRB2 +9/-9 polymorphism were 32% (-9/-9), 49% (+9/-9) and 19% (+9/+9), and 19% (II), 53% (ID), 28% (DD) for the ACE I/D polymorphism. The baseline and training characteristics of runners are summarized in the Table 1 for the ACE I/D polymorphism and in the Table 2 for the BDKRB2 +9/-9 polymorphism. There are no significant differences in general characteristics between the genotypes.

**TABLE 1:** Baseline and training characteristics of marathon runners classified by ACE I/D polymorphism.

ACE I/D	DD	ID	II
Age (years)	39.4 ± 6.1	40.1 ± 6.9	44.7 ± 6.1
Weight (kg)	76.9 ± 8.4	75.6 ± 11.5	74.6 ± 8.4
Height (m)	1.74 ± 0.1	1.72 ± 0.1	1.76 ± 0.05
BMI (kg/m^2^)	25.3 ± 1.8	24.7 ± 5.1	24.1 ± 2.7
% Fat mass	21.7 ± 4.5	22.0 ± 4.9	20.8 ± 4.7
Fat mass (kg)	16.6 ± 4.7	17.0 ± 5.5	15.7 ± 4.5
Free fat mass (kg)	59.4 ± 5.7	58.8 ± 6.6	58.8 ± 5.8
TE (years)	6.4 ± 3.5	7.7 ± 6.2	8.5 ± 5.2
10 km race (minutes)	46.0 ± 6.7	46.8 ± 5.8	46.3 ± 3.3
Speed Peak (km/h)	18.3 ± 2.0	18.5 ± 2.0	18.6 ± 1.7
VO_2_ peak (ml/kg/min)	52.4 ± 6.6	54.2 ± 9.6	52.9 ± 5.9
Race time (minutes)	250.5 ± 45.1	261.0 ± 44.2	255.3 ± 31.4

BMI, body mass index; TE, training experience. The values presented are the mean ± SD, of 14 runners (II), 39 runners (ID), 21 runners (DD).

**TABLE 2:** Baseline and training characteristics of marathon runners classified by BDKRB2 +9/-9 polymorphism.

BDKRB2 +9/-9	-9/-9	+9/-9	+9/+9
Age (years)	42.9 ± 7.6	39.8 ± 5.7	41.5 ± 7.6
Weight (kg)	76.1 ± 9.4	75.9 ± 8.9	75.5 ± 12.7
Height (m)	1.73 ± 0.05	1.74 ± 0.1	1.73 ± 0.1
BMI (kg/m^2^)	25.4 ± 3.0	24.4 ± 4.9	25.2 ± 3.2
% Fat mass	22.4 ± 4.1	21.4 ± 4.7	22.8 ± 5.7
Fat mass (kg)	17.2 ± 4.7	16.4 ± 4.8	17.6 ± 6.8
Free fat mass (kg)	58.8 ± 5.9	59.5 ± 6.1	57.9 ± 7.5
TE (years)	5.9 ± 3.1	6.9 ± 3.9	8.1 ± 3.3
10 km race (minutes)	48.2 ± 6.2	45.8 ± 5.5	48.2 ± 6.2
Speed Peak (km/h)	17.8 ± 2.1	18.7 ± 2.2	18.1 ± 1.7
VO_2_ peak (ml/kg/min)	55.3 ± 14.4	53.1 ± 7.1	51.3 ± 7.3
Race time (minutes)	254.9 ± 34.6	257.4 ± 46.5	267.5 ± 41.4

BMI, body mass index; TE, training experience. The values presented are the mean ± SD, of 24 runners (-9/-9), 36 runners (+9/-9) and 14 runners (+9/+9).

### Exercise-Induced Cytokines in Different Genotypes of the BDKRB2 Polymorphism

**FIGURE 1:**
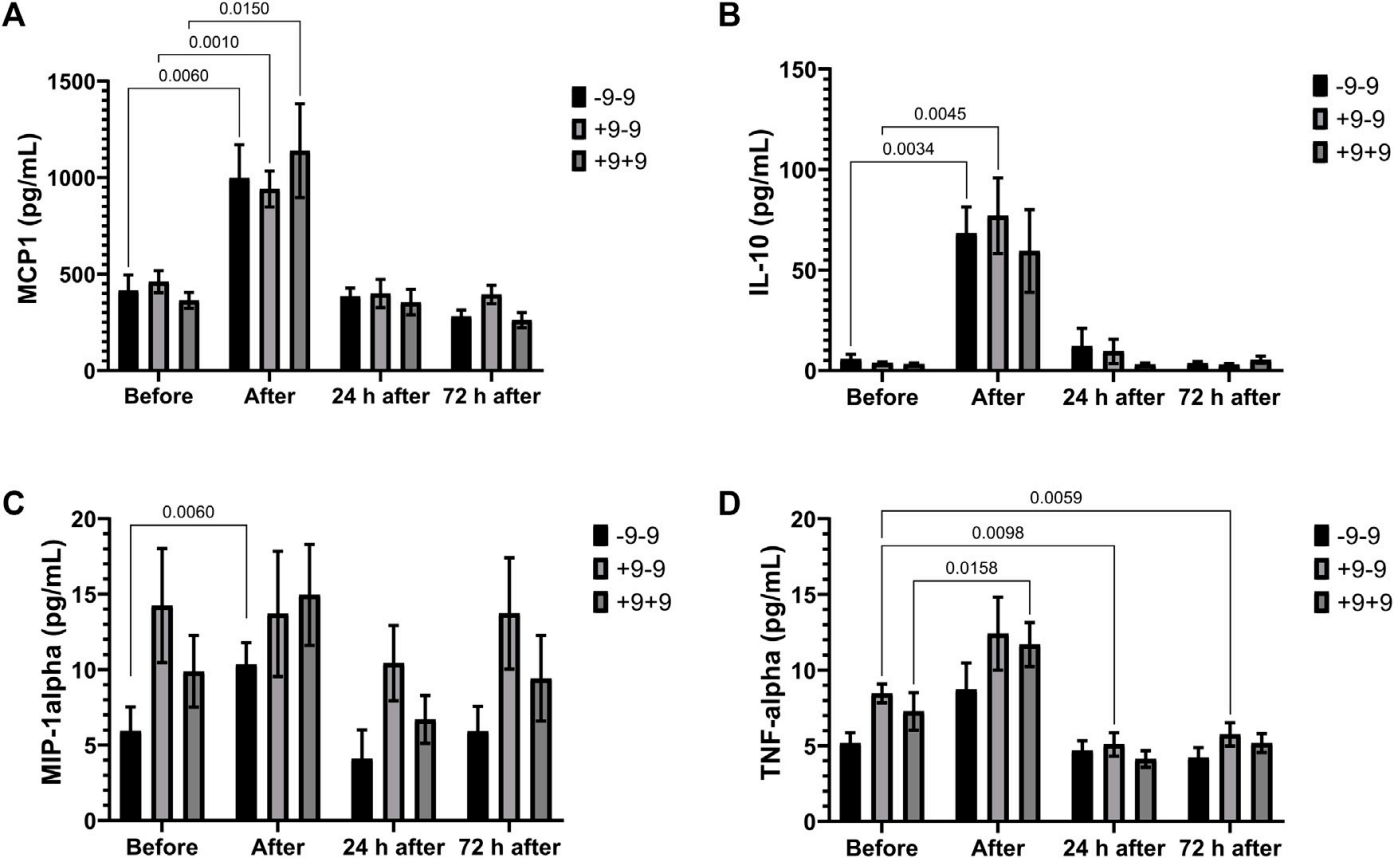
Inflammatory mediators in -9/-9, +9/-9, +9/+9 genotypes. Plasma concentrations of MCP-1 **(A)**, IL-10 **(B)**, MIP-1alpha **(C)** and TNF-alpha **(D)** before, immediately after, 24 and 72 h after the race were determined. Values are presented as mean and standard error of the mean of 11 runners with genotype -9/-9, 18 runners with +9/-9 runners, and 12 runners with genotype +9/+9. Comparisons between genotypes and time were performed by two-way repeated measures ANOVA and Sidak’s multiple comparisons test.

Marathon race promotes an elevation of MCP-1 concentration in all genotypes (Figure 1A) and of IL-10 levels in the -9 allele carriers (-9/-9 and +9/-9 genotypes) and of MIP1-alpha in -9/-9 homozygotes (Figures 1B,C). On the other hand, TNF-a increased significantly in the +9/+9 homozygotes after the race, as well as a reduction of TNF-a in the +9/-9 heterozygotes in the recovery period (Figure 1D). IL-8 tended to increase after the race in all genotypes but not significantly (data not shown).

**FIGURE 2:**
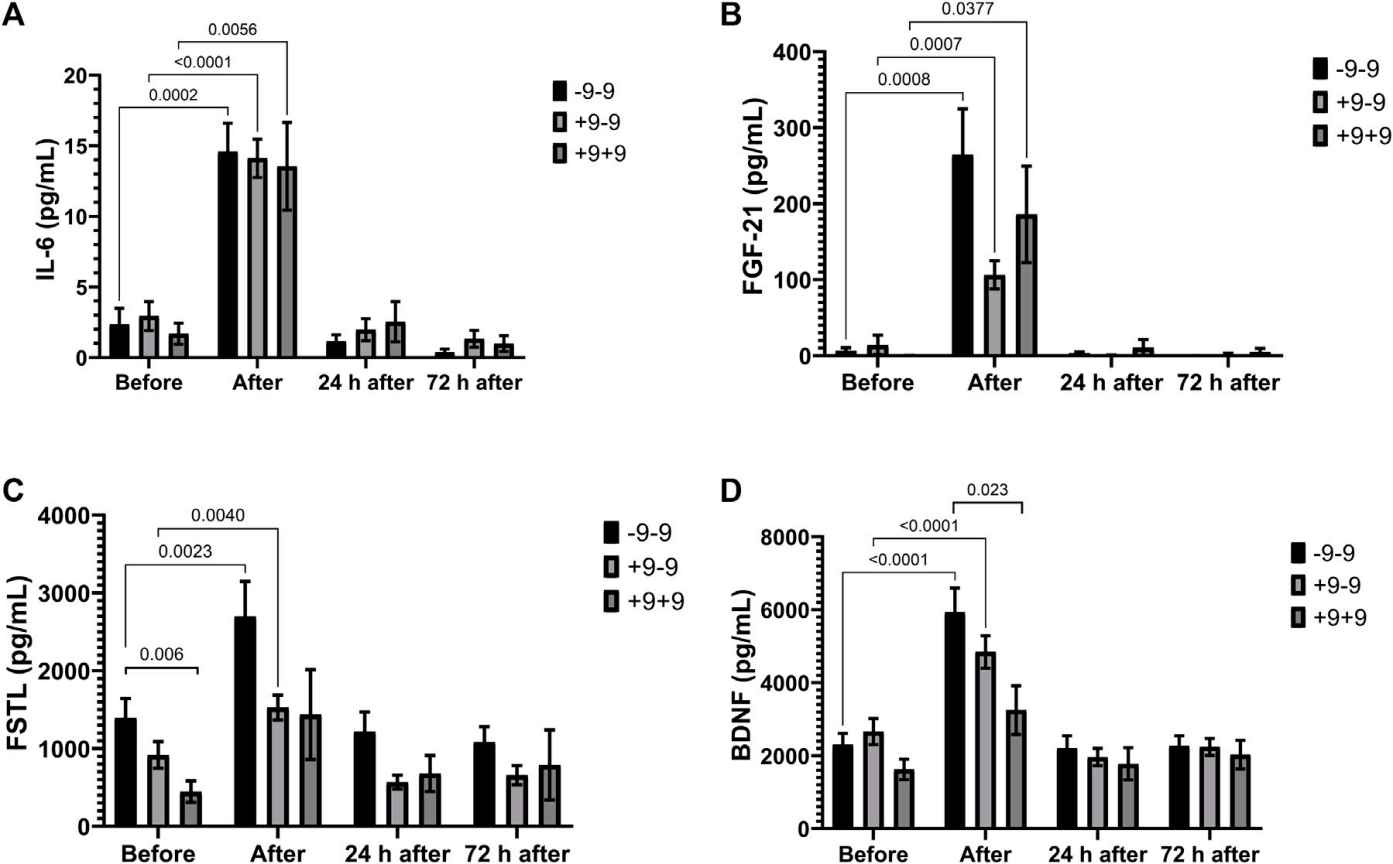
Exercise-induced cytokines upregulated in -9/-9, +9/-9, +9/+9 genotypes. Plasma concentrations of IL-6 **(A)**, FGF-21 **(B)**, FSTL **(C)** and BDNF **(D)** before, immediately after, 24 and 72 h after the race were determined. Values are presented as mean and standard error of the mean of 24 runners (-9/-9), 36 runners (+9/-9) and 14 runners (+9/+9). Comparisons between genotypes and time were performed by two-way repeated measures ANOVA and Sidak’s multiple comparisons test.

Marathon induced an increase in IL-6 and FGF-21 in all genotypes (Figures 2A,B). Plasma concentrations of FSTL and BDNF increased significantly after the race in -9 allele carriers (Figures 2C,D). FSTL and BDNF concentrations were higher in the −9/−9 homozygotes compared to the +9/+9 homozygotes before and after the race, respectively (Cohen d = 1.16; effect-size r = 0.5 and Cohen d = 0.98; r = 0.44, respectively) (Figures 2C,D).

**FIGURE 3:**
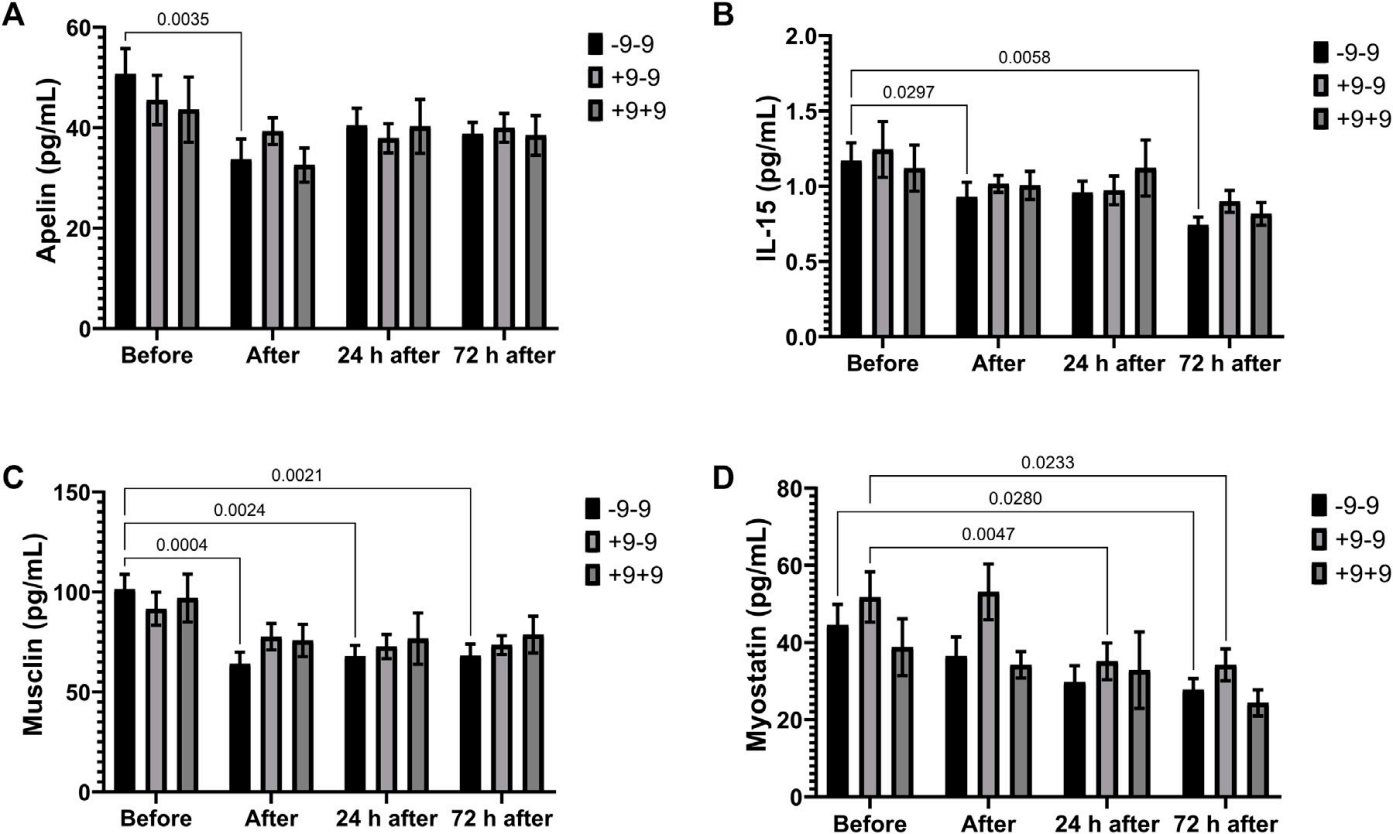
Exercise-induced cytokines downregulated in -9/-9, +9/-9, +9/+9 genotypes. Plasma concentrations of apelin **(A)**, IL-15 **(B)**, musclin **(C)** and myostatin **(D)** before, immediately after, 24 and 72 h after the race were determined. Values are presented as mean and standard error of the mean 24 runners (-9/-9), 36 runners (+9/-9) and 14 runners (+9/+9). Comparisons between genotypes and time were performed by two-way repeated measures ANOVA and Sidak’s multiple comparisons test.

Apelin (immediately after the race), IL-15 (immediately and 24 h after the race) and musclin (immediately, 24 and 72 h after the race) concentrations were significantly reduced after the marathon only in the -9/-9 homozygotes (Figures 3A–C). The concentration of myostatin reduced in +9/-9 genotypes (24 and 72 h after the race) and -9/-9 genotypes (24 h after the race) (Figure 3D).

### Exercise-Induced Cytokines in Different Genotypes of the ACE I/D Polymorphism

**FIGURE 4:**
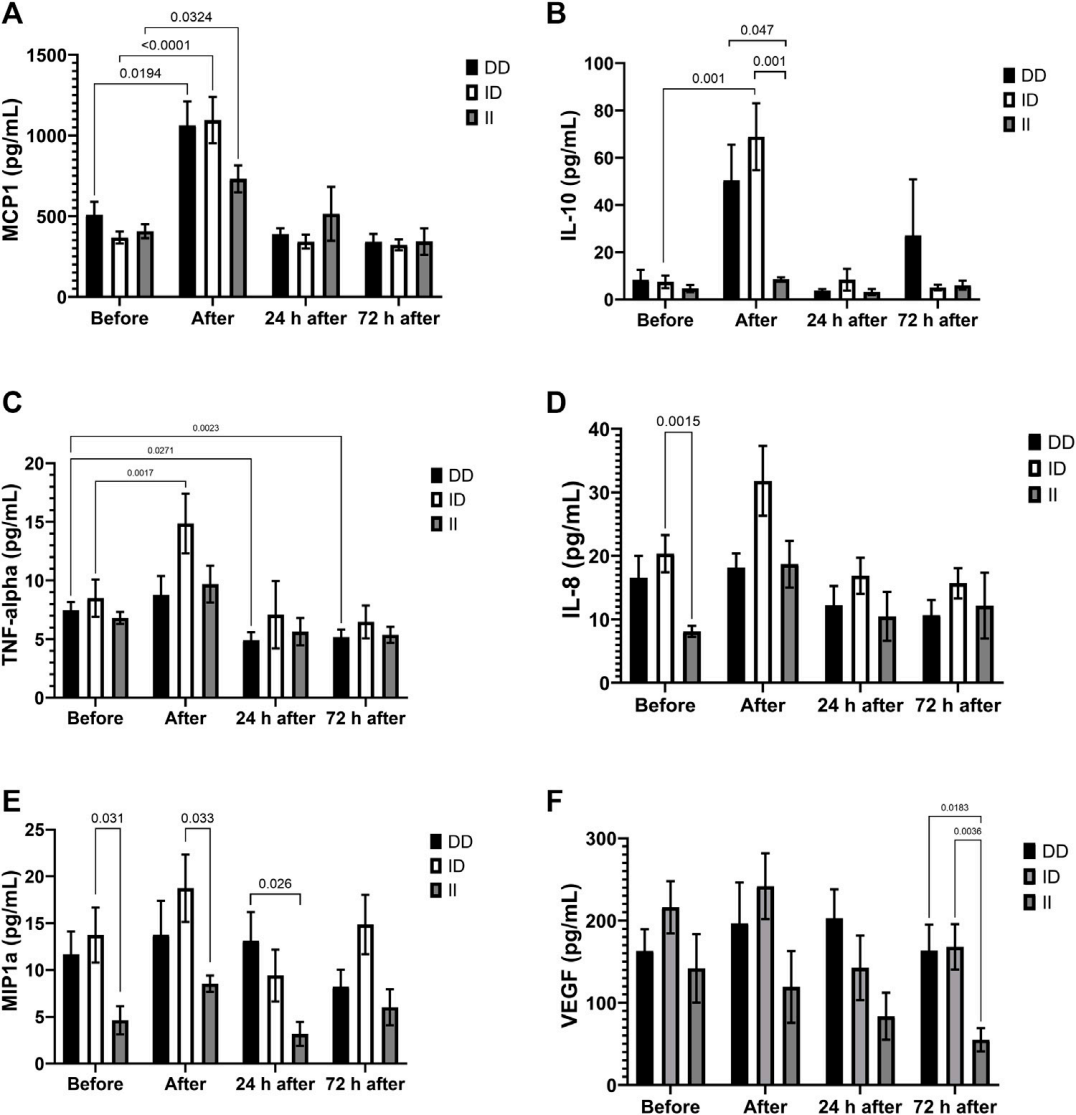
Inflammatory mediators in II, ID and DD genotypes. Plasma concentrations of MCP-1 **(A)**, IL-10 **(B)**, IL-8 **(C)**, MIP-1alpha **(D)**, TNF-alpha **(E)** and VEGF **(F)** before, immediately after, 24 and 72 h after the race were determined. Values are presented as mean and standard error of the mean of seven runners with genotype II, 22 runners with ID runners, and 14 runners with genotype DD. Comparisons between genotypes and time were performed by two-way repeated measures ANOVA and Sidak’s multiple comparisons test.

Marathon increased plasma concentrations of MCP-1 in all genotypes and of IL-10 and TNF-alpha in runners with ID genotype (Figures 4A–C). Plasma concentrations of IL-8 and MIP-1alpha before the race (Figures 4D and 4E), of MIP-1alpha and IL-10 after the race (Figures 4A,E) and VEGF 72 h after the race (Figures 4F) were lower in II homozygotes compared to runners with the presence of D allele. One day after the race we also observed lower levels of MIP-1alpha in runners with II homozygotes compared to DD homozygotes (Figures 4E). TNF-alpha levels decreased 24 h and 72 h after the race in DD homozygotes (Figures 4C). The Cohen’s d and effect-size r from comparison of cytokines in the genotypes of ACE I/D polymorphism are described on Table 3, suggesting large to very large effect-size.

**FIGURE 5:**
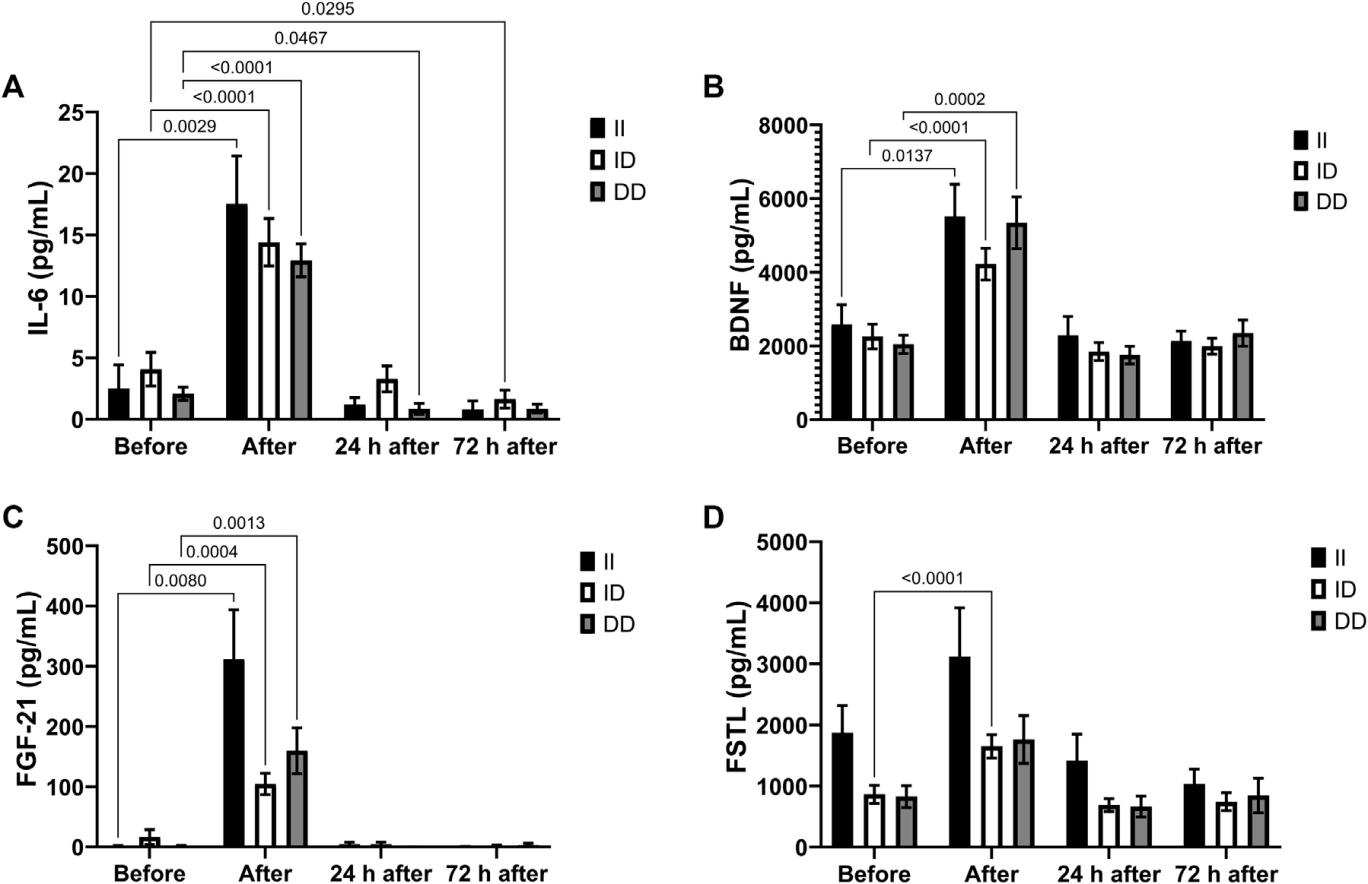
Exercise-induced cytokines upregulated in II, ID and DD genotypes. Plasma concentrations of IL-6 **(A)**, BDNF **(B)**, FGF-21 **(C)** and FSTL **(D)** before, immediately after, 24 and 72 h after the race were determined. Values are presented as mean and standard error of the mean of 14 runners (II), 39 runners (ID), 21 runners (DD). Comparisons between genotypes and time were performed by two-way repeated measures ANOVA and Sidak’s multiple comparisons test.

The plasma concentrations of IL-6, BDNF and FGF-21 increased in all genotypes after the race and IL-6 decreased in the recovery period only in the DD genotype group (24 h after) and ID genotype group (72 h after) (Figures 5A–C). There was a significant increase in plasma FSTL concentration only in runners with the ID genotype (Figure 5D).

**FIGURE 6:**
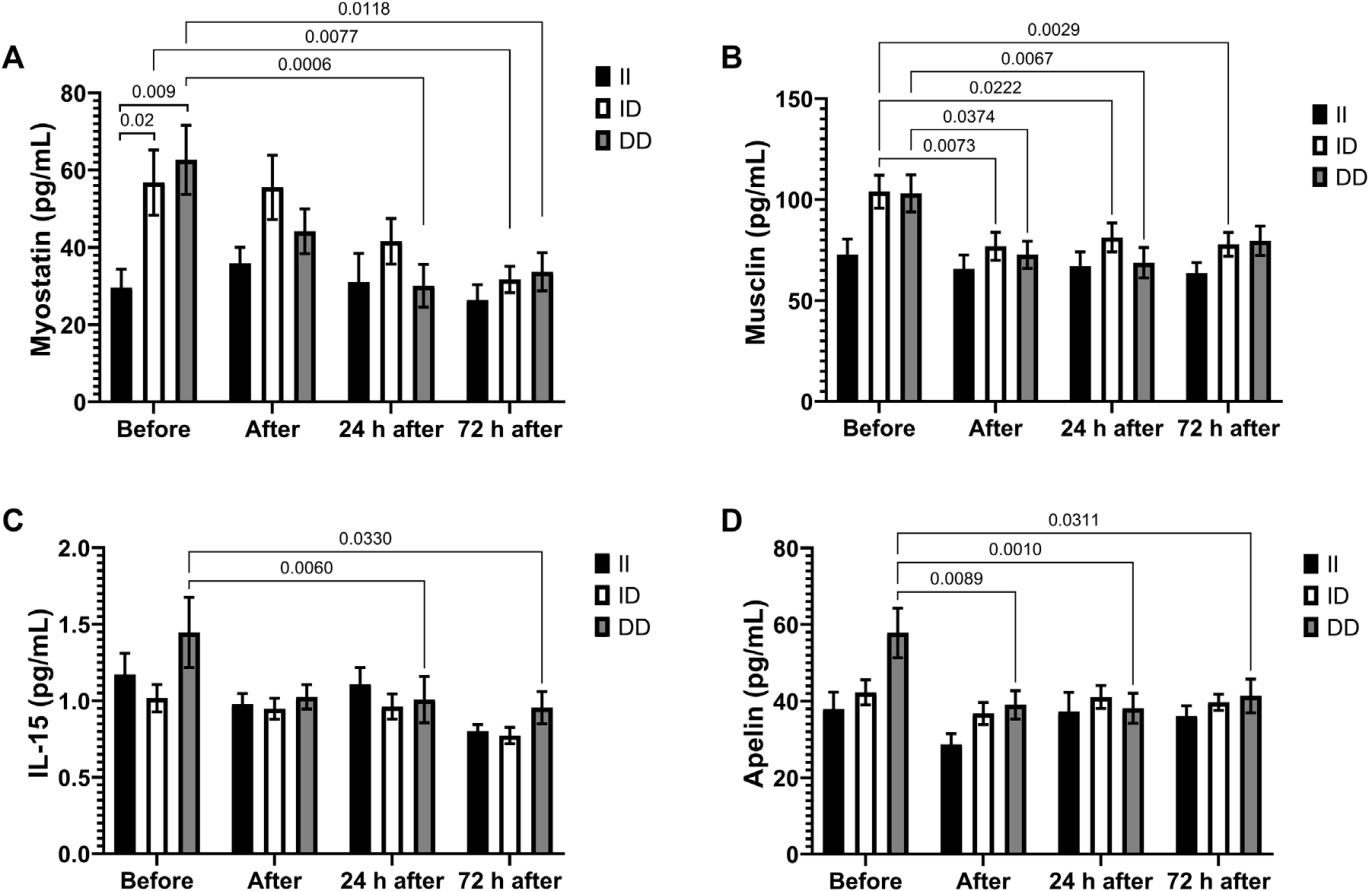
Exercise-induced cytokines downregulated in II, ID and DD genotype. Plasma concentrations of myostatin **(A)**, musclin **(B)**, IL-15 **(C)** and apelin **(D)** before, immediately after, 24 and 72 h after the race were determined. Values are presented as mean and standard error of the mean of 14 runners (II), 39 runners (ID), 21 runners (DD). Comparisons between genotypes and time were performed by two-way repeated measures ANOVA and Sidak’s multiple comparisons test.

Plasma myostatin and musclin levels decreased after race in the D alleles carriers (Figures 6A,B), as well as IL-15 and apelin plasma levels in the DD homozygotes (Figures 6C,D). Before the race myostatin concentrations were higher in DD homozygotes compared to II homozygotes (Figure 6A).

### ACE Activity

**FIGURE 7:**
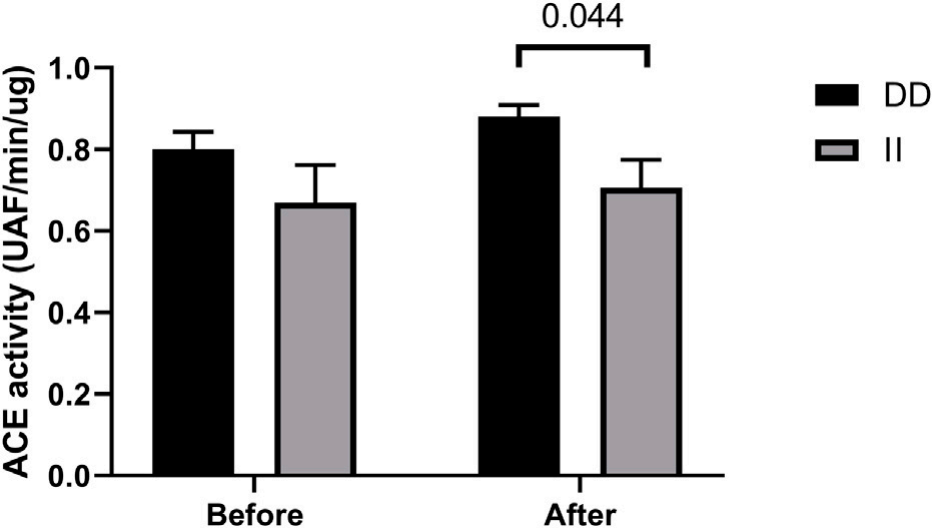
Angiotensin converting enzyme (ACE) activity in II and DD homozygotes. ACE activity before and immediately after the race were determined. Values are presented as mean and standard error of the mean of 12 runners (II) and 13 runners (DD). Comparisons between genotypes and time were performed by Sidak’s multiple unpaired *t*-test.

ACE activity tended to increase after the race in runners with DD genotypes (vs. before) and was higher before the race in DD homozygotes compared to II homozygotes, but not significantly. Moreover, DD homozygotes had higher ACE activity compare to II homozygotes after the race (*p* < 0.044) (Figure 7).

**FIGURE 8:**
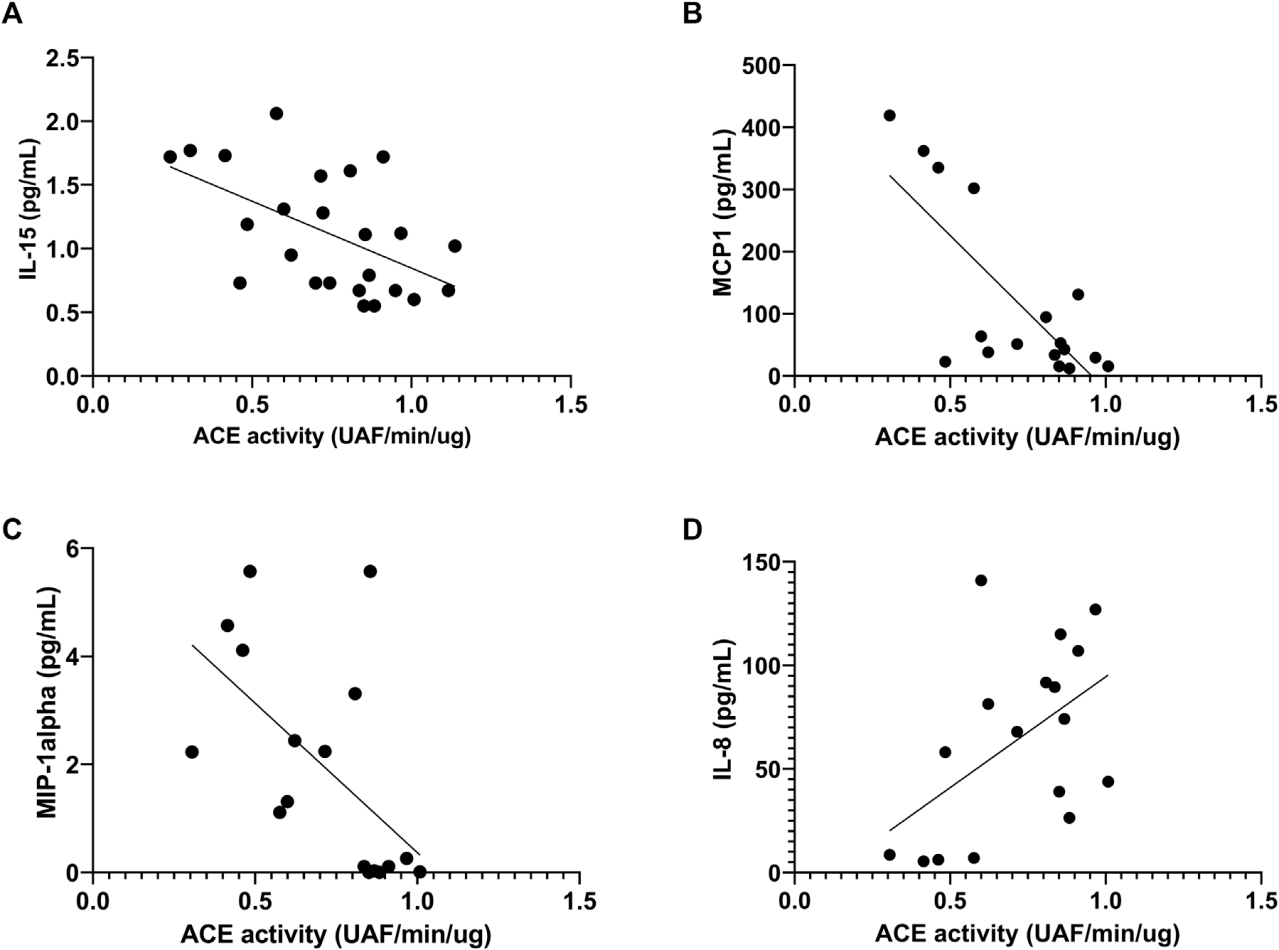
Correlation between Angiotensin converting enzyme (ACE) activity and cytokines before race. Correlations between ACE activity and IL-15 **(A)**, MCP-1 **(B)**, MIP-1alpha **(C)** and IL-8 **(D)** levels were performed in 25 runners by Spearman.

**FIGURE 9:**
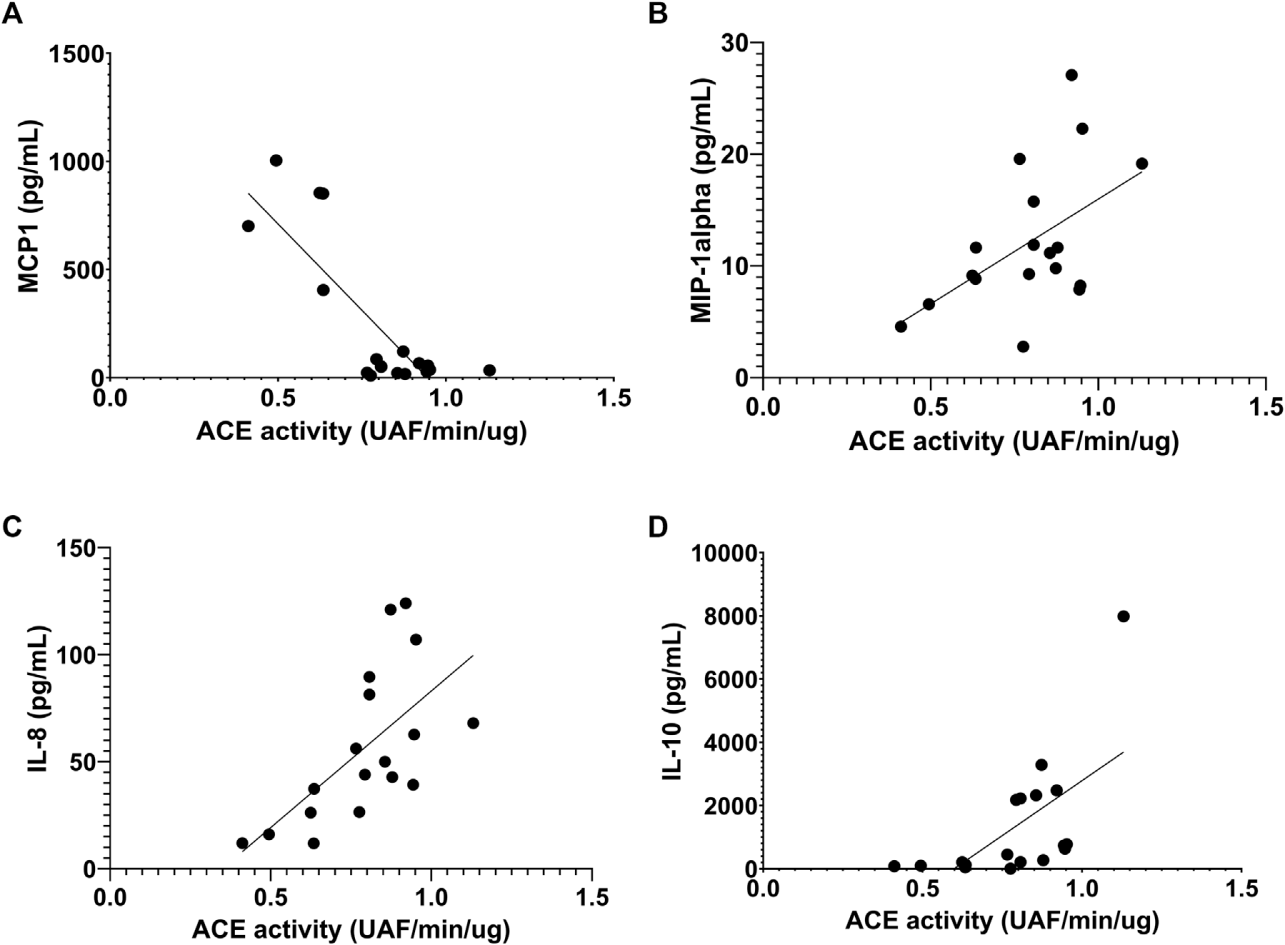
Correlation between Angiotensin converting enzyme (ACE) activity 606 and cytokines after the race. Correlations between ACE activity and MCP-1 **(A)**, MIP-1alpha **(B)**, IL-8 **(C)** and IL-10 **(D)** levels were performed in 25 runners homozygotes (II or DD) by Spearman.

Before the race, we observed negative correlation of ACE activity with pro-inflammatory cytokines, IL-15, MCP1 and MIP-1alpha, r = -0.55, *p* < 0.047; r = -64, *p* < 0.0073 and r = -0.63, *p* < 0.0075, respectively (Figures 8A–C) and a positive correlation with IL-8, r = 0.49, *p* <0.046 (Figure 8D). After the race ACE activity was also negatively correlated with pro-inflammatory cytokine MCP-1, r = -56, *p* < 0.016 (Figure 9A) and positively correlated with IL-8, IL-10 and MIP1-alpha, r = 0.72, *p* < 0.0007, r = 0.72, p < 0.0007 and r=0.47, *p* < 0.048, respectively (Figures 9B–D).

## Discussion

The runners with the presence of -9 allele have greater response in exercise-induced cytokines such as IL-10, FSTL, BDNF involved on muscle recovery process as well as higher changes of apelin, IL-15, musclin and myostatin levels. Runners with D allele has higher levels of pro-inflammatory cytokines (MIP-1alpha, TNF-alpha) before and after race as well higher levels of IL-10 after race, an anti-inflammatory mediators, and higher myostatin levels before race. The runners with the presence of D allele significantly reduce myostatin and musclin, IL-15, IL-6 and apelin levels after race**.**

In the classical pathway of RAS, renin promotes the formation of Ang I from AGT and ACE catalysis the formation of Ang II from Ang I, which can bind to AT1 and AT2 receptors promoting opposite biological effects. The interaction with AT1R causes vasoconstriction, increased sympathetic tone, promotes cell proliferation, inflammation, fibrosis and angiogenesis. Conversely, the interaction of Ang II with AT2R promotes vasodilation, decrease of sympathetic tonus and anti-inflammatory, antiproliferative and antiangiogenic properties (Lau et al., 2020; Bekassy et al., 2021). In muscle tissue, Ang II by binding to AT1R may inhibit IGF-1-AKT-mTOR pathway and activate NOX2-dependent ROS production, consequently NfκB pathway impairing muscle remodeling (Yamamoto et al., 2020). However, Ang II also could be converted in Ang one to seven, which acts on MAS receptor leading to vasodilation, anti-inflammatory and proangiogenic response. In the muscle adaptations to exercise, Ang II is associated with smooth muscle growth in vessels, increase capillary density, hypertrophy, increased oxygen consumption and regular contraction excitation process (Bellamy et al., 2010). We observed higher anti- and pro-inflammatory response (MIP1-α, IL-8 and IL-10) after race in runners with D allele compared to II homozygotes suggesting that ACE I/D polymorphism may modulate the inflammatory process induced by exercise and pro-inflammatory response follow anti-inflammatory response after exercise seems to be crucial to tissues repair and regeneration.

DD homozygotes had higher ACE activity compare to II homozygotes after the race. ACE activity were positively correlated with anti-inflammatory cytokines indicating that the anti-inflammatory response in the runners with D allele is associated to higher ACE activity which may acts on AT2R. However, the pro-inflammatory response of D allele not be attributed by ACE activity because the ACE activity was negatively correlated to pro-inflammatory mediators before and after the race. The activation of AT1R and AT2R after exercise and in the recovery period should be investigated. VEGF is a pro-angiogenic factor stimulated by Ang II, and we also observed higher levels of VEGF in runners with the presence of D allele compared to II homozygotes 72 h after the race.

Previous study of our group demonstrated a reduction of myostatin 3 days after the race which may contribute to muscle repair after exercise and a decrease of apelin and musclin in the same period (de Sousa et al., 2021). The role of these changes on muscle repair remains unclear and should be investigated. Before the race the levels of myostatin were higher as well as IL-15, apelin and musclin tended to be higher, but not significantly, in runners with DD genotypes compared to II genotypes. After the race we observed a decrease of myostatin, IL-15, apelin and musclin in runners with DD genotypes reaching levels similar of runners with II genotypes. These cytokines remained unchanged in runners with II genotypes. We suggest that ACE I/D polymorphism influences myostatin levels and consequently, protein homeostasis. DD genotype has been associated to left ventricular hypertrophy (Fatini et al., 2000; Kasikcioglu et al., 2004; Di Mauro et al., 2010), however, lower metabolic response of skeletal muscle in athletes (Valdivieso et al., 2017).

RAS and KKS have various possibilities of interaction to modulate vascular tone and inflammation. ACE degrades bradykinin, which is the first interaction described between these systems. Moreover, AT1R and AT2R can form heterodimers with B2R and increase NO (nitric oxide) production by endothelial cells, as well as B2R activation induces increased renin expression (Lau et al., 2020; Arazi et al., 2021; Bekassy et al., 2021).

Herein, we demonstrated greater IL-10 response and higher levels of BDNF in runners with -9 allele. IL-10 is the mainly anti-inflammatory mediator that triggers the swift of M1 to M2, which is crucial to modulate the differentiation and fusion of myogenic cells on muscle regeneration (Molina et al., 2021). BDNF is an abundant neurotrophic factor produced and released by brain and skeletal muscle in response to exercise (Neeper et al., 1995; Wang et al., 2019) and the cellular sources of BDNF include muscle fiber type II, satellites cells and endothelial cells of skeletal muscle (Matthews et al., 2009; Rasmussen et al., 2009; Delezie et al., 2019; Di Rosa et al., 2021; Cefis et al., 2022). BDNF activates tropomyosin-related kinase B (TrkB) receptors which triggers a complex set of signaling cascades including PI3-K/MAPK pathways and seems to promote fat oxidation via AMPK kinase and acetyl coenzyme A carboxylase-beta (ACC-beta); stimulates PGC-1α expression which is involved in mitochondrial biogenesis; modulates autophagy in response to damage (Mousavi and Jasmin, 2006; Matthews et al., 2009; Pedersen et al., 2009; Rasmussen et al., 2009; Lee and Jun, 2019; Di Rosa et al., 2021) and activates the proliferation of satellite cells leading to an improvement of muscle repair/regeneration (Omura et al., 2005; Clow and Jasmin, 2010; Di Rosa et al., 2021). This molecule also has been associated to cardioprotection by reduction on cardiomyocyte apoptosis and mitochondrial dysfunction (Hang et al., 2021).

We also observed higher FSTL and lower myostatin levels in runners with -9 allele after race, which contributes to muscle mass and repair. Myostatin was the first myokine described on literature and FSTL is a potential myokine target induced by exercise (Domin et al., 2021). Myostatin acts on the activin receptors (type I and II), promoting the phosphorylation and activation of small mothers against decapentaplegic (SMAD) proteins. SMAD-2 and SMAD-3 form a complex with SMAD-4, which induces the transcription of catabolic genes, promote protein degradation through the ubiquitin-proteasome system and autophagy (Hoffmann and Weigert, 2017; Lee and Jun, 2019; Piccirillo, 2019), and are fully regulated by FSTL (Chen et al., 2021). This molecule may be stimulated by fibro-adipogenic progenitors after muscle injury (Molina et al., 2021) and has myogenic properties that directly inhibits myostatin from binding to the activin IIb receptor and suppression of SMAD-3 phosphorylation, consequently increasing protein synthesis by the mTOR/S6K/S6RP signaling cascade (Winbanks et al., 2012; Hoffmann and Weigert, 2017; Lee and Jun, 2019). FSTL also is considered a hepatokine and cardiokine associated to cardioprotection with properties to improve endothelial function and revascularization after injury (Severinsen and Pedersen, 2020; Domin et al., 2021).

In summary, we can conclude that BDKRB2 +9/-9 polymorphisms seem to influence exercise-induced cytokines related to muscle regeneration and/or cardioprotection such as IL-10, BDNF, FSTL and myostatin, highlighting an important role of B2R on exercise adaptation. In addition, ACE I/D polymorphisms may influence myostatin levels and consequently protein homeostasis as well the inflammatory process contributing to tissue repair. The molecular mechanism of RAS and KKS regulating directly or indirectly IL-10, BDNF, FSTL or myostatin levels should be more carefully investigated.

## Data Availability

The original contributions presented in the study are included in the article/supplementary materials, further inquiries can be directed to the corresponding author.
